# Unprecedently large ^37^Cl/^35^Cl equilibrium isotopic fractionation on nano-confinement of chloride anion

**DOI:** 10.1038/s41598-022-05629-6

**Published:** 2022-02-02

**Authors:** Mateusz Pokora, Agata Paneth, Piotr Paneth

**Affiliations:** 1 International Center for Research on Innovative Biobased Materials (ICRI-BioM) – International Research Agenda, Lodz University of Technology, Żeromskiego 116, 90-924 Lodz, Poland; 2 Department of Organic Chemistry, Faculty of Pharmacy, Medical University of Lublin, Chodźki 4a, 20-093 Lublin, Poland; 3 Institute of Applied Radiation Chemistry, Faculty of Chemistry, Lodz University of Technology, Żeromskiego 116, 90-924 Lodz, Poland

**Keywords:** Physical chemistry

## Abstract

Confinement can result in unusual properties leading to new, exciting discoveries in the nano-realm. One such consequence of confinement at the nanoscale is extremally large isotopic fractionation, especially at sub-van der Waals distances. Herein, on the example of chlorine isotope effects, we show that at conditions of nanoencapsulation these effects may reach values by far larger than observed for the bulk environment, which in the case of nanotubes can lead to practical applications (e.g., in isotopic enrichment) and needs to be considered in analytical procedures that employ nanomaterials.

## Introduction

Many physicochemical properties change dramatically at the nanoscale compared to bulk properties. A number of these differences originate in the confinement of the molecules in nano or sub-nano structures. Sulfur allotropy^1^, change in electrochemical reactivity^2^, and ferroelectric properties of polymers in narrow nanotubes^3^, solar cell operations^4^, liquid crystals properties in microdroplets^5^, hydrogen atom transfer in micelles^6^, or glass transitions in mesoporous nanochannels^7^, are among recently described examples of such phenomena. Confinement, by influencing molecular vibrations^8–10^, can also lead to isotopic fractionation. While the change of properties of water confined in nanotubes has been documented some time ago^11^ it is only recently that the related deuterium isotope effects have been reported^12,13^. Kinetics and equilibrium deuterium isotope effects on the confinement at the macroscale have also been observed in the past^14–17^. They are, however, much larger than heavy-atom^18^ isotope effects which have neither been measured nor predicted thus far (although they were recently reported for conductivity^19^ and diffusion^20^).

Chlorine isotopic fractionation is a very informative tool used in many life-controlling processes^21^, including environmental^22–25^, geochemical^26–30^ and biochemical^31–33^ studies. Therefore a detailed understanding of this phenomenon is an important scientific (but with practical consequences in the interpretation of experimental results^34^ and analytical techniques^35–37^) issue. It is thus not surprising that studies on model reactions^38^ and theoretical predictions have been performed^33,34,39–41^. In our recent studies^42^, we have shown that noncovalent chlorine isotope effects can be correlated with the hydrogen bond strength. However, even when hydrogen bonds were approaching a very weak region the isotope effects, albeit small, did not disappear. We have, therefore, attempted to investigate the sources of this “residual” chlorine isotopic fractionation with the assumption that it originates in van der Waals interactions, and following our studies of the adsorption on the graphene^43^, we have initially considered chloride anion adsorbed on graphene. The isotope effect was not alleviated completely, which prompted us to study isotopic consequences of chloride interactions with other nanostructures. We have observed that encapsulation leads to values by far larger than any value observed at the bulk scale where the largest chlorine kinetic isotope effects are expected not to exceed about 25 ‰ (expressed as the deviation from unity, see Eq. (2))^41^. In this contribution, we present the results of chlorine isotope effects of chloride trapped in different nanostructures and show their surprisingly large values and their relation to the distance to the surrounding environment. For comparison, micro-solvated chloride in nanotubes, as well as boron-nitrogen^44^ and gold^45^ cages have been considered.

## Theoretical methods

Geometries of all considered structures have been first optimized in the gas phase to the nearest energy minimum at the DFT level of theory, using ωB97X-D functional^46^ (which includes G2 Grimme dispersion correction for all atoms treated explicitly) expressed in the def2-TZVP basis set^47^ as implemented in the Gaussian16 program^48^. Default convergence criteria have been applied. They are available in the Supplementary Information. Vibrational analysis has been used to ensure that the optimized geometry corresponds to a stationary point representing a minimum on the potential energy surface (3n-6 real vibrations). The influence of the inclusion of the counterpoise correction^49^ for the basis set superposition error (BSSE) has been found to be negligible (Table 2). SMD Polarized Continuum Model of solvent with parameters for the aqueous solution (which includes dispersion correction in the CDS part that applies to the bulk properties of the solvent) has been used^50^. Chlorine equilibrium isotope effects, ^37^Cl-EIE, were calculated at 298 K according to the Bigeleisen equation which relates an isotope effect to vibrational frequencies^51^:

1 $${\text{EIE}} = {{\mathop \prod \limits_{{\text{i}}}^{{3{\text{n}}_{{\text{R}}} - 6}} \frac{{{\text{u}}_{{{\text{Ri}}}}^{{\text{H}}} \cdot {\text{sinh}}\left( {\frac{{{\text{u}}_{{{\text{Ri}}}}^{{\text{L}}} }}{2}} \right)}}{{{\text{u}}_{{{\text{Ri}}}}^{{\text{L}}} \cdot {\text{sinh}}\left( {\frac{{{\text{u}}_{{{\text{Ri}}}}^{{\text{H}}} }}{2}} \right)}}} \mathord{\left/ {\vphantom {{\mathop \prod \limits_{{\text{i}}}^{{3{\text{n}}_{{\text{R}}} - 6}} \frac{{{\text{u}}_{{{\text{Ri}}}}^{{\text{H}}} \cdot {\text{sinh}}\left( {\frac{{{\text{u}}_{{{\text{Ri}}}}^{{\text{L}}} }}{2}} \right)}}{{{\text{u}}_{{{\text{Ri}}}}^{{\text{H}}} \cdot {\text{sinh}}\left( {\frac{{{\text{u}}_{{{\text{Ri}}}}^{{\text{H}}} }}{2}} \right)}}} {\mathop \prod \limits_{{\text{i}}}^{{3{\text{n}}_{{\text{P}}} - 6}} \frac{{{\text{u}}_{{{\text{Pi}}}}^{{\text{L}}} \cdot {\text{sinh}}\left( {\frac{{{\text{u}}_{{{\text{Pi}}}}^{{\text{H}}} }}{2}} \right)}}{{{\text{u}}_{{{\text{Pi}}}}^{{\text{L}}} \cdot {\text{sinh}}\left( {\frac{{{\text{u}}_{{{\text{Pi}}}}^{{\text{H}}} }}{2}} \right)}}}}} \right. \kern-\nulldelimiterspace} {\mathop \prod \limits_{{\text{i}}}^{{3{\text{n}}_{{\text{P}}} - 6}} \frac{{{\text{u}}_{{{\text{Pi}}}}^{{\text{H}}} \cdot {\text{sinh}}\left( {\frac{{{\text{u}}_{{{\text{Pi}}}}^{{\text{L}}} }}{2}} \right)}}{{{\text{u}}_{{{\text{Pi}}}}^{{\text{L}}} \cdot {\text{sinh}}\left( {\frac{{{\text{u}}_{{{\text{Pi}}}}^{{\text{H}}} }}{2}} \right)}}}}$$

in which R and P denote reactant and product, respectively, n is the number of atoms, u_i_ = hν_i_/k_B_T, where h and k_B_ are Planck and Boltzmann constants, respectively, T is absolute temperature, and ν_i_ are the frequencies of normal modes of vibrations. Calculations were performed using harmonic frequencies with the aid of the Isoeff program^52^.

## Results and discussion

**Figure 1:**
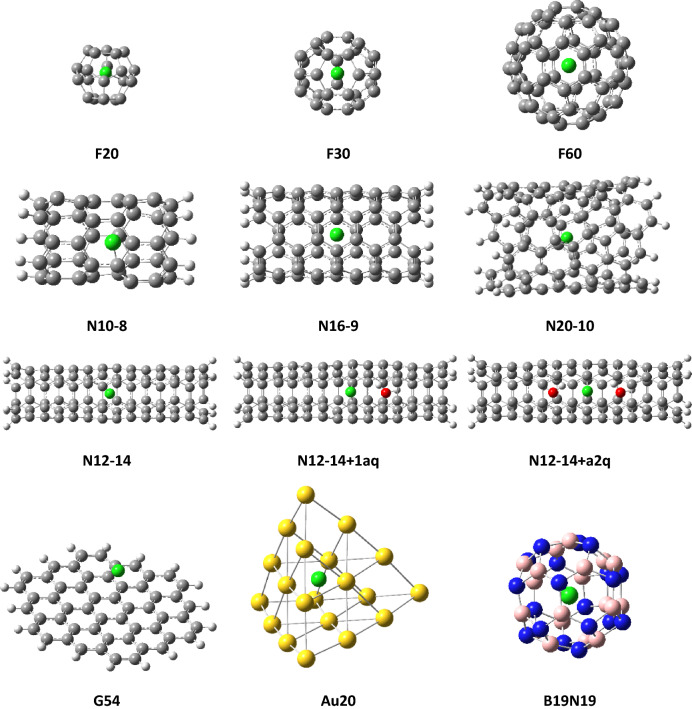
Nanostructures used in modeling of chlorine isotope effects.

To find out the influence of confinement five nanotubes of different lengths and radius, and three different fullerenes were studied. The graphene sheet was used as the reference that does not impose any confinement. The studied structures are collected in Table 1 which also introduces symbols used; the letter indicates the type; G—graphene, N—nanotubes, and F—fullerene. For G and F types the number corresponds to the number of carbon atoms. In the case of nanotubes, the first digits represent the number of carbon atoms in the cross-section and thus provide information on the structure diameter while the second defines the length of the model (compare **N12-7**, **N12-10**, and **N12-14** in Fig. 1). Additionally, we have tested the influence of the type of elements that form the cage. For this purpose, we used a tetrahedral gold pyramid of 20 atoms and a fullerene-type cage formed by 19 nitrogen and 19 boron atoms.

**Table 1:** Minimal X-Cl (X = C, N, B, or Au) distances, equilibrium isotope effects and isotopic fractionations.

Structure	Minimal Distance, Å	^37^Cl-EIE	ε^37^Cl, ‰
F20	1.65	0.95700	44.9
F30	2.03	0.97412	26.6
F60	3.52	0.99769	2.3
N10-8	1.85	0.97469	25.0
N12-7	1.73	0.97148	29.4
N12-10	1.73	0.97174	29.1
N12-14	1.75	0.97189	28.9
N12-14 + 1aq	1.79	0.97447	26.2
N12-14 + 2aq	1.80	0.97220	28.6
N16-9	2.77	0.98952	10.6
N20-10	3.78	0.99953	0.5
B19N19	2.50	0.98610	14.1
Au20	2.73	0.99527	4.8
G54	3.21	0.99941	0.6

Since chlorine equilibrium isotope effects, ^37^Cl-EIE, are very small, the results are presented as isotopic fractionation factors, ε, which express an isotope effect as the deviation from unity in “per-mil” units [‰] (that correspond to mUr of the SI system):

2 $$\varepsilon^{{{37}}} {\text{Cl}} = \left[ {{1}/^{{{37}}} {\text{Cl-EIE}}{ - } { 1}} \right]*1000$$

In this notation, negative values correspond to isotope effects larger than unity (so-called normal isotope effects) while positive values correspond to isotope effects smaller than unity (so-called inverse isotope effects).

**Figure 2:**
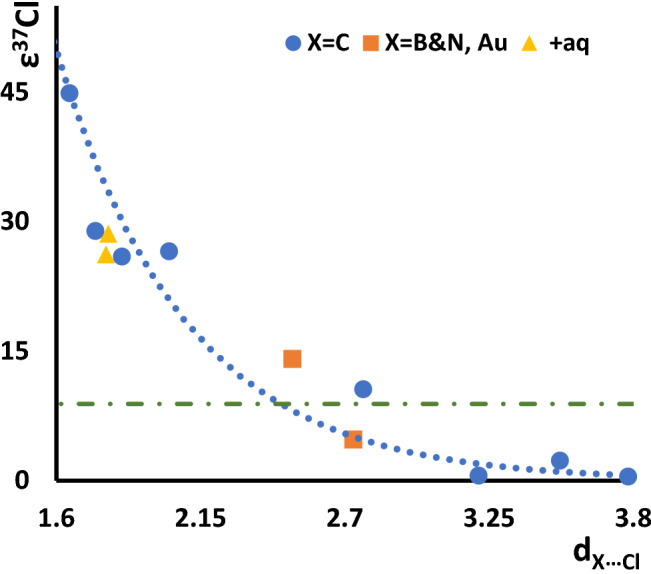
Dependence of the chlorine isotopic fractionation (ε^37^Cl in ‰) on the distance (d_X_…_Cl_ in Å) of chloride from the nearest atom of the nanostructure. Blue circles refer to the distance from the carbon atom. Yellow triangles refer to the distance from the carbon atom in cases with continuum models of solvent included. Distances from Au and B are represented by orange squares (see Table 1 for numerical values). The green line at about 9 ‰ corresponds to the maximum equilibrium isotope effect.

**Figure 3:**
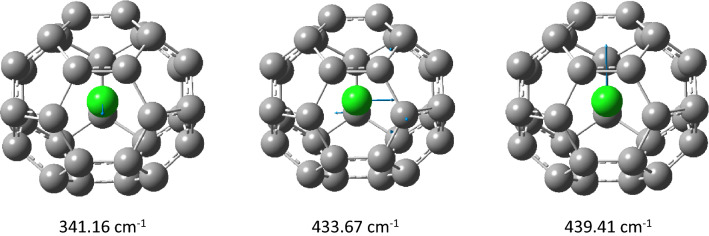
Displacement vectors of the three most isotope-sensitive vibrations.

We have employed hybrid density functional from the family that has been shown successful in modeling non-covalent interactions in the recent benchmark studies^53^. The obtained results for the equilibrium between chloride anion in the gas phase and nano-environment are illustrated in Fig. 3, which represents the dependence of ε^37^Cl on the distance between chloride anion and nearest atom of the nano-structure. The corresponding numerical results are collected in Table 1. As can be seen, it increases exponentially (the dotted blue line illustrates this trend) when the distance becomes smaller. The green line in this figure corresponds to ^37^Cl-EIE on the putative equilibrium between chloride ion and its incorporation into a C–Cl covalent bond in the gas phase, which marks the maximum equilibrium isotope effect in the bulk of about 9 ‰ (the green line in Fig. 2). Within this limit, only ε^37^Cl of complexes without serious spatial constraints are contained (e.g., **F60**). For chloride confined in small fullerenes (e.g., **F30**) or narrow nanotubes (e.g., **N10**), the distances to the closest nanostructure are small and the resulting isotopic fractionation reaches values significantly larger than those encountered in a bulk environment. In fact, in the absolute sense, they are larger than the values of the largest expected chlorine kinetic isotope effects (about 24.3 ‰^41^). Even larger isotopic fractionation is observed when confinement results in covalent interactions (e.g., **F20**).

Isotope effects and thus corresponding isotopic fractionations arise from differences in isotopic vibrations. We have analyzed contributions from individual frequencies to the overall calculated EIE on the examples representing different nanostructures (graphene, **G54**, nanotube, **N16-9**, and fullerene, **F30**) and the whole range of the isotopic fractionation (compare entries in Table 1). In all cases, only a few frequencies exhibit a shift upon substitution of ^35^Cl by ^37^Cl, with three vibrations along three coordinate axes involving chloride displacement exhibiting the largest isotopic shift as illustrated by Fig. 3 on the example of **F30**. These frequencies, together with corresponding force constants are collected in Table 2 (complete lists of isotopic frequencies for these structures are provided in the Supplementary Information, Tables S15–S17).

**Table 2:** Vibrational analysis of normal modes associated with chlorine atom and its nearest carbon neighbor.

Structure	Atom	Frequencies/Force constants			Cl–C and C–C distances		
**G54**	Cl	11.30/0.00_2_	21.71/0.00_7_	104.03/0.07	3.21		
	C(9)	395.70/0.51	759.94/2.09	1453.8/12.21	1.42_3_	1.40_3_	1.40_5_
**G54 no Cl**^**−**^		773.31/3.07	774.80/2.12	1449.49/12.71	1.42_5_	1.40_6_	1.40_7_
**N16-9**	Cl	63.90/0.07	296.22/0.90	296.54/0.90	2.77		
	C(23)	594.13/1.13	684.75/2.98	1291.16/9.92	1.43_8_	1.43_5_	1.41
**N16-9 no Cl**^**−**^		727.46/1.97	1509.17/13.82	1614.77/17.90	1.43_6_	1.43_6_	1.39
**F30**	Cl	341.16/1.59	433.67/1.84	439.41/2.54	2.03		
	C(16)	90.92/0.06	560.44/2.25	1249.16/11.04	1.53	1.48	1.48
**F30 no Cl**^**−**^		728.02/3.75	1280.09/11.59	1348.99/12.87	1.45	1.44	1.44

Frequencies in cm^−1^, force constants in mdyne/Å, distances in Å. Numbering of carbon atoms corresponds to structures provided in the respective table of the Supplementary Information.

Additionally, frequencies and force constants associated with vibrations involving the carbon atom closest to the chloride and its distances to the neighboring carbon atoms are listed. A comparison of the properties with the structure without the chloride is also provided in Table 2. As can be seen from the comparison of the last two rows the C–C distances of the carbon atom that is closest to the chloride anion are shorter indicating that encapsulation leads to a swelling of the nanostructure. The vibrational pattern in which this atom participates also changes, however, these modes are not isotope sensitive so they do not affect the isotopic fractionation.

Thus far we have considered isotopic fractionation on the equilibrium between chloride anion isolated in the gas phase and the nano-environment. Equally, or maybe even more important, is the transfer from the condensed phase, in particular from the aqueous solution since the nano-environment has a significant influence on the properties of water and solvation^54,55^. In such cases, the values reported in Table 2 and Fig. 3 are smaller by about 4 ‰ which corresponds to the chlorine isotopic fractionation on the transfer of chloride anion from the gas phase to the aqueous solution^42^. On the example of **N16-9** we show, however, that the use of the continuum solvent model underestimates the effect of the polar environment, leading to the ε^37^Cl value lower by only about 1 ‰ – compare entries in the first and third raw of Table 3.

**Table 3:** Influence of counterpoise BSSE correction and continuum solvent model.

Property	Minimal distance, Å	^37^Cl-EIE	ε^37^Cl [‰]
Gas phase	2.77176	0.98952	10.59
Counterpoise	2.77178	0.98958	10.53
Implicit PCM SMD	2.77181	0.99057	9.52

To study the effect of micro-solvation in nanotubes one or two water molecules have been added to the **N12-14** model. For these studies, it was necessary to use the elongated nanostructures to confine water molecules within the hydrophobic environment of the nanotube. As expected, and evidenced by values collected in Table 3, the elongation of the nanotube has a negligible influence on the isotopic fractionation (compare results for **N12-7**, **N12-10**, and **N12-14** in Table 3). The obtained values indicate that micro-solvation has also a negligible effect on chlorine isotopic fractionation and does not alleviate its enormous enhancement caused by the confinement.

**Figure 4:**
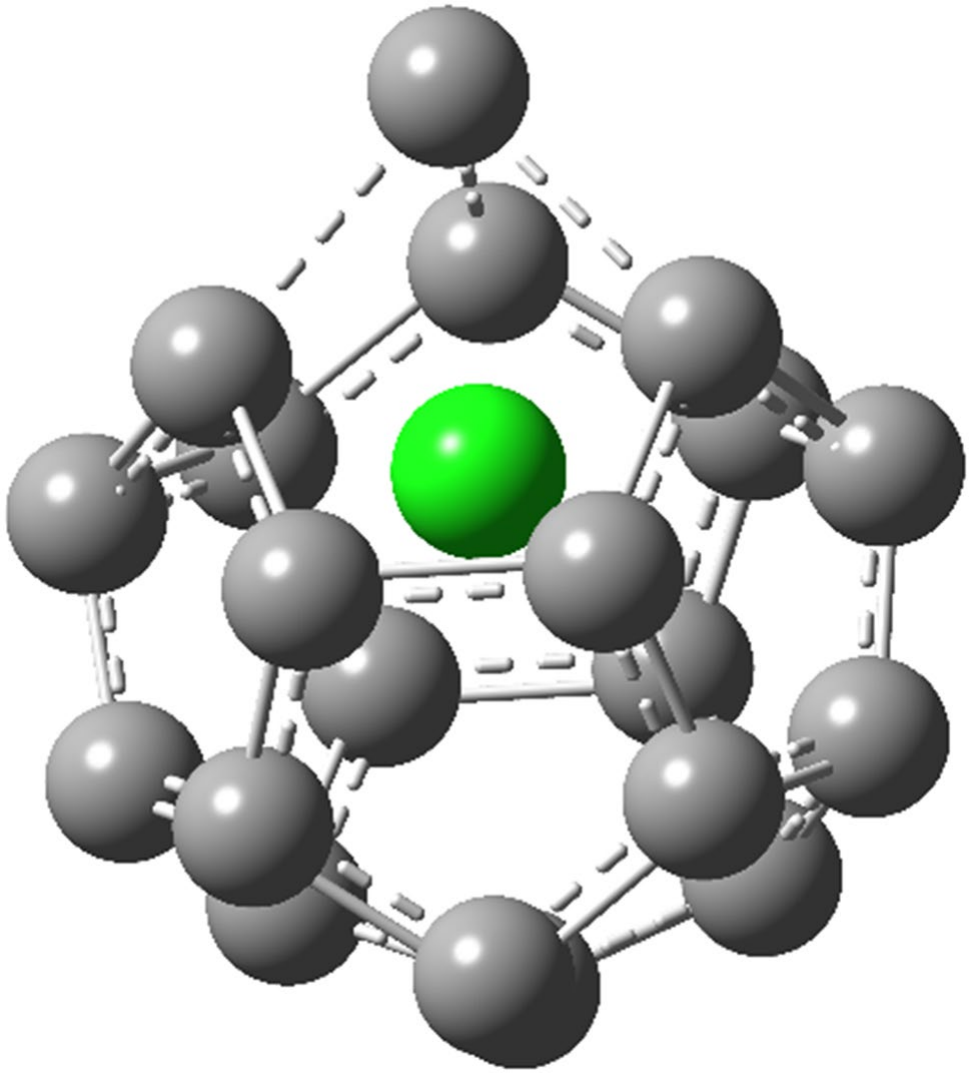
Structure of chloride encapsulated in **F20**.

The extreme case of **F20** deserves additional analysis. In this case, chloride does not occupy the center of the nanostructure but is shifted 0.6 Å toward the edge, which causes elongation of bonds to the carbon atom which is pushed out by about 0.45 Å as illustrated by Fig. 4. The chloride anion position is stiffened by interactions with three neighboring carbon atoms, which are at practically covalent distances (1.65 Å). Thus the source of this huge isotopic fractionation goes beyond a simple effect of encapsulation.

Finally, energy aspects need to be considered. Structures in which chloride is caged within a nanostructure, i.e., systems other than nanotubes, are of no practical relevance regarding their exploitation for isotopic enrichment since the encapsulation energy is high and the formation of these structures requires the opening of a “window” in the nanostructure^56,57^, which usually requires harsh conditions and about 80 kcal/mol^58^ although lower energies might suffice in the case of functionalized structures.

## Conclusions

The most important conclusion of the present studies is, exemplified by the calculations on chloride anion in nano-environment, extremely large isotopic fractionation caused by the confinement in constrained structures. Furthermore, solvation with even such polar solvents as water has only a minor effect on this phenomenon. This observation can lead to new methods of isotopic enrichment, especially for systems/elements which exhibit small isotopic fractionation under bulk conditions. More importantly, it also calls for special caution in the interpretation of experimental protocols of purification of material for isotopic ratio measurements as well as procedures used in these analyses.

## Supplementary Information

Supplementary Tables.

## Data Availability

The optimized structures used in this study are available in Supplementary Tables S1 to S14. Tables S15 to S17 provide isotopic frequencies for structures **F30**, **N16-9**, and **G54**, respectively.
