# Supplementary material for: Unprecedently large 37Cl/35Cl equilibrium isotopic fractionation on nano-confinement of chloride anion
Source: Sci Rep. 2022 Feb 2;12:1768. doi: 10.1038/s41598-022-05629-6 (PMC8811032; doi:10.1038/s41598-022-05629-6)
Supplement: Supplementary file 1 — Supplementary Tables. [file 41598_2022_5629_MOESM1_ESM.pdf]

Supplementary Tables

Unprecedently large $^{37}Cl/^{35}Cl$ equilibrium isotopic fractionation on nano-confinement of chloride anion

Mateusz Pokora, Agata Paneth and Piotr Paneth*

Corresponding author: piotr.paneth@p.lodz.pl

Contents:

Table S1. XYZ coordinates of the N16-9 structure. 1
Table S2. XYZ coordinates of the F20 structure. 2
Table S3. XYZ coordinates of the F30 structure. 2
Table S4. XYZ coordinates of the F60 structure. 2
Table S5. XYZ coordinates of the N10-8 structure. 3
Table S6. XYZ coordinates of the N12-10 structure. 3
Table S7. XYZ coordinates of the N12-7 structure. 4
Table S8. XYZ coordinates of the N12-14 structure. 5
Table S9. XYZ coordinates of the N12-14+1aq structure. 6
Table S10. XYZ coordinates of the N12-14+2aq structure. 7
Table S11. XYZ coordinates of the N20-10 structure. 8
Table S12. XYZ coordinates of the G54 structure. 9
Table S13. XYZ coordinates of the B19N19 structure. 10
Table S14. XYZ coordinates of the Au20 structure. 11
Table S15. F30 isotopic frequencies and shifts (in cm^{-1}). 11
Table S16. N16-9 isotopic frequencies and shifts (in cm^{-1}). 12
Table S17. G54 isotopic frequencies and shifts (in cm^{-1}). 15

Table S1. XYZ coordinates of the N16-9 structure.

C	-4.887409	-2.674928	-0.870733
C	-4.887380	2.009892	-1.968293
C	-2.441213	-1.880465	-2.015715
C	-3.668991	-1.280078	-2.411479
C	3.669029	2.729233	0.075857
C	2.441336	2.641215	0.789680
C	1.229858	2.767646	0.087081
C	1.229856	2.440942	-1.306879
C	2.441315	2.015735	-1.880456
C	3.669052	2.411549	-1.280122
C	0.000026	2.649218	0.815984
C	0.000026	2.011169	-1.907309
C	-1.229785	2.441007	-1.306922
C	-1.229789	2.767726	0.087089
C	-2.441213	2.015735	-1.880472
C	-2.441237	0.789691	-2.641213
C	-1.229788	0.087100	-2.767699
C	0.000026	0.816019	-2.649193
C	1.229857	0.087091	-2.767619
C	2.441338	0.789677	-2.641206
C	2.441312	1.880446	2.015729
C	1.229847	1.306882	2.440937
C	0.000024	1.907326	2.011141
C	-1.229780	1.306923	2.441002
C	-2.441214	1.880459	2.015725
C	-2.441236	2.641221	0.789692
C	-3.668988	2.411502	-1.280087
C	-3.668966	2.729173	0.075842
C	-3.668971	0.075842	-2.729165
C	-1.229782	-1.306912	-2.440983
C	1.229850	-1.306870	-2.440919
C	0.000024	-1.907307	-2.011175
C	-4.887405	0.870729	-2.674913
C	-4.887401	2.674927	0.870729
C	-4.887388	1.968287	2.009877
C	-3.668995	1.280086	2.411493
C	-3.668969	-0.075834	2.729166
C	-2.441231	-0.789671	2.641204
C	-1.229782	-0.087075	2.767733
C	1.229845	-0.087066	2.767646
C	0.000022	-0.815990	2.649162
C	3.669055	1.280123	2.411546
C	3.669026	-0.075847	2.729228
C	2.441325	-0.789657	2.641196
C	4.887454	2.674917	0.870722
C	4.887440	1.968271	2.009880
C	4.887436	2.009886	-1.968275
C	4.887460	0.870715	-2.674902
C	3.669034	0.075853	-2.729225
C	3.669052	-1.280119	-2.411530
C	2.441310	-1.880452	-2.015719
C	-4.887403	-0.870723	2.674912
C	4.887439	-1.968271	-2.009874
C	-2.441240	-2.641234	-0.789693
C	0.000022	-2.649217	-0.816024
C	-1.229788	-2.767756	-0.087101
C	-3.668974	-2.729169	-0.075849
C	-4.887388	-1.968281	-2.009876
C	-4.887375	-2.009884	1.968294
C	-3.668986	-2.411487	1.280075
C	-2.441205	-2.015724	1.880448
C	-1.229778	-2.441050	1.306917
C	0.000022	-2.011209	1.907328
C	1.229840	-2.440979	1.306871
C	2.441299	-2.015725	1.880431
C	4.887450	-0.870714	2.674906
C	4.887422	-2.009884	1.968282
C	3.669042	-2.411537	1.280113
C	4.887457	-2.674923	-0.870722
C	3.669031	-2.729231	-0.075862
C	2.441335	-2.641227	-0.789680
C	1.229851	-2.767670	-0.087093
H	-5.805121	-2.564838	1.818925
H	5.805196	-2.564775	1.818848
H	5.805225	-3.105715	-0.490364
H	-5.805150	-3.105804	-0.490406
H	-5.805129	2.564840	-1.818912
H	-5.805147	0.490402	-3.105788
H	-5.805139	3.105808	0.490402
H	-5.805142	1.818897	2.564814
H	5.805221	3.105711	0.490366
H	5.805220	1.818816	2.564753
H	5.805212	2.564768	-1.818828
H	5.805229	0.490358	-3.105691
H	-5.805144	-0.490407	3.105798
H	5.805219	-1.818808	-2.564747
H	-5.805139	-1.818880	-2.564814
H	5.805218	-0.490370	3.105708
Cl	-0.000882	-0.000016	0.000018

Table S2. XYZ coordinates of the F20 structure.

C	0.502332	-1.805408	-1.014908	C	-0.798578	1.660649	0.973843
C	0.502474	-2.004079	0.522316	C	-0.798805	1.853767	-0.519517
C	-0.797575	-1.674150	0.951298	C	0.501156	1.781957	-1.056261
C	-1.683930	-1.399164	-0.180812	C	1.443325	1.442173	0.186330
C	-0.797723	-1.377391	-1.345882	C	0.501412	1.454688	1.474618
C	1.443841	-0.558860	-1.341281	C	2.676684	-0.000338	-0.000266
C	1.443909	-0.881651	1.154907	C	-1.684515	0.542040	1.301405
C	-0.798018	-0.477246	1.865455	C	-1.684725	0.855204	-1.120729
C	-0.797967	0.012773	-1.925202	C	0.501761	0.550118	-1.997126
C	-2.046396	-0.000788	-0.000063	Cl	0.659807	0.000604	0.000172
C	0.501885	0.023994	2.071387				

Table S3. XYZ coordinates of the F30 structure.

C	1.409038	-2.024170	0.689571	C	-2.479723	-0.001366	1.130424
C	0.694137	-2.394593	-0.537791	C	-2.436514	-1.162834	0.292244
C	-0.693969	-2.394620	-0.537797	C	-1.408935	-2.024229	0.689535
C	-1.219771	-1.524296	-1.657351	C	0.000010	0.767398	-2.009144
C	-2.246878	-0.736211	-1.100657	C	-1.219878	1.528618	-1.654739
C	-2.246327	0.737872	-1.098763	C	-0.694275	2.395666	-0.532977
C	-2.436633	1.161477	0.294055	C	0.694141	2.395701	-0.532962
C	-1.408943	2.022674	0.693308	C	1.408779	2.022752	0.693348
C	-0.696397	1.333221	1.829395	C	0.000096	-0.761042	-2.008966
C	0.696264	1.333292	1.829430	C	0.696205	-1.336682	1.827620
C	1.396257	-0.001872	2.007634	C	-0.696183	-1.336696	1.827607
C	2.479706	-0.001226	1.130498	C	2.436526	1.161606	0.294110
C	2.436580	-1.162703	0.292310	C	2.246329	0.738001	-1.098698
C	2.246955	-0.736081	-1.100573	C	1.219834	1.528690	-1.654723
C	1.219932	-1.524259	-1.657355	Cl	-0.000009	-0.000752	-0.122206
C	-1.396336	-0.001955	2.007661				

Table S4. XYZ coordinates of the F60 structure.

C	-2.119427	-1.742312	2.207843	C	-1.127281	-0.477114	-3.301684
C	-2.578871	-2.213956	0.922334	C	-1.069910	-1.777886	-2.845283
C	-1.583945	-3.120157	0.397651	C	0.208936	-2.392562	-2.575343
C	-0.509523	-3.208519	1.358924	C	1.373131	-1.679141	-2.774093
C	-0.840469	-2.356918	2.477611	C	2.447431	-1.767674	-1.812696
C	0.150204	-1.637581	3.113802	C	2.309708	-2.565868	-0.695709
C	-0.091430	-0.269805	3.509697	C	2.769323	-2.094337	0.589903
C	-1.313030	0.317428	3.251960	C	3.346251	-0.845806	0.700945
C	-2.350322	-0.435653	2.586278	C	3.015072	0.005750	1.819609
C	-3.051259	0.460622	1.696557	C	2.954684	1.367392	1.341448
C	-3.490234	0.010208	0.468487	C	2.004823	2.233463	1.842891
C	-3.248462	-1.357598	0.072558	C	1.303419	3.129409	0.953253
C	-2.954669	-1.367487	-1.341496	C	-0.064649	3.227858	1.406009
C	-2.004784	-2.233595	-1.842909	C	-1.091055	3.312682	0.487758
C	-1.303374	-3.129572	-0.953269	C	-0.797320	3.302506	-0.926411
C	0.064666	-3.227986	-1.406002	C	-1.834532	2.549581	-1.592422

C	1.091045	-3.312769	-0.487717	C	-1.518273	1.736117	-2.661084
C	0.797321	-3.302671	0.926424	C	-0.150217	1.637405	-3.113712
C	1.834544	-2.549737	1.592456	C	0.091433	0.269660	-3.509528
C	1.518280	-1.736241	2.661082	C	1.313122	-0.317575	-3.252131
C	2.122075	-0.429333	2.777168	C	2.350287	0.435557	-2.586297
C	1.127288	0.476984	3.301701	C	3.051266	-0.460730	-1.696563
C	1.069930	1.777719	2.845238	C	3.490318	-0.010311	-0.468519
C	-0.208916	2.392415	2.575297	C	3.248537	1.357539	-0.072580
C	-1.373135	1.679033	2.774059	C	2.578963	2.213858	-0.922352
C	-2.447464	1.767605	1.812746	C	1.583980	3.119942	-0.397651
C	-2.309685	2.565769	0.695747	C	0.509501	3.208189	-1.358853
C	-2.769345	2.094241	-0.589902	C	0.840526	2.356759	-2.477618
C	-3.346255	0.845723	-0.700982	C	2.119521	1.742271	-2.208023
C	-3.015102	-0.005847	-1.819640	Cl	-0.000191	0.001360	0.000090
C	-2.122106	0.429207	-2.777221				

Table S5. XYZ coordinates of the N10-8 structure.

C	2.837429	0.186074	1.603976	C	1.481594	-1.532364	-1.586388
C	3.512644	-1.137103	1.371810	C	0.714896	-0.228487	-1.947228
C	1.508296	0.238532	1.963198	C	-1.458379	-1.483586	-1.532682
C	0.656035	-1.056835	1.939878	C	-0.783362	-0.275013	-2.051437
C	-1.478976	0.257686	1.878628	C	-2.867558	-1.510449	-1.483242
C	-0.750045	-1.003208	1.815063	C	-3.597600	-0.291949	-1.732977
C	-2.901376	0.265766	1.669216	C	2.613718	-2.214167	1.022135
C	-3.599690	-0.911874	1.545061	C	3.460267	-1.978446	-0.116013
C	2.631151	2.375360	0.422610	C	1.294014	-2.078241	1.035397
C	3.508508	1.408674	1.037180	C	0.663791	-1.937267	-0.342640
C	1.308244	2.259267	0.422640	C	-1.452936	-1.928008	0.852952
C	0.688767	1.475315	1.568443	C	-0.775382	-2.110875	-0.414070
C	-1.332973	2.204584	0.412721	C	-2.862514	-1.932393	0.819525
C	-0.687678	1.433407	1.539786	C	-3.514938	-2.169241	-0.407992
C	-2.642586	2.413161	0.450868	H	4.486343	-1.314295	1.818585
C	-3.543070	1.525855	1.149613	H	-4.666393	-0.854895	1.364303
C	2.831755	0.979441	-1.632025	H	4.480634	1.693501	1.429673
C	3.485129	1.876107	-0.614794	H	-4.477471	1.867128	1.583093
C	1.475901	1.113928	-1.891285	H	4.445855	2.325859	-0.839903
C	0.673432	1.972414	-0.934481	H	-4.477187	2.331822	-0.797052
C	-1.488547	0.922360	-1.698709	H	4.601355	-0.187982	-1.482328
C	-0.698375	1.909579	-0.921881	H	-4.665776	-0.304314	-1.551205
C	-2.911333	0.867646	-1.479835	H	4.405244	-2.499926	-0.225295
C	-3.545663	1.846703	-0.523784	H	-4.597324	-2.123553	-0.396965
C	2.832483	-1.350445	-1.334988	Cl	0.435024	-0.116597	-0.085924
C	3.559813	-0.227108	-1.781281				

Table S6. XYZ coordinates of the N12-10 structure.

C	-5.480894	2.168398	-0.415093	C	1.905024	1.949074	0.938333
C	-5.464914	2.023985	0.911158	C	3.096364	1.485472	1.499075
C	-4.246697	1.423497	1.481775	C	3.085180	0.148974	2.052637

C	-4.238842	0.159350	1.992872	C	1.880806	-0.561083	1.975451
C	-5.459948	-0.660328	2.071970	C	1.880168	-1.778012	1.173211
C	-5.480469	-1.742795	1.289932	C	3.089988	-2.084631	0.535570
C	-4.272101	-2.007019	0.495608	C	3.078285	-1.891028	-0.915275
C	-4.256737	-1.810168	-0.863293	C	1.879862	-1.500704	-1.496322
C	-5.486083	-1.428897	-1.586249	C	1.878289	-0.134034	-2.057346
C	-5.500294	-0.210063	-2.122769	C	3.085920	0.581625	-1.986814
C	-4.279103	0.584547	-1.907475	C	3.101897	1.758486	-1.162841
C	-4.275872	1.685052	-1.117234	C	4.360063	1.996536	-0.482020
C	-3.006083	2.085591	-0.499831	C	4.354005	1.844808	0.876577
C	-2.988204	1.894264	0.909080	C	5.561012	1.445881	1.616859
C	-1.769124	1.546703	1.540649	C	5.546322	0.221092	2.151335
C	-1.778136	0.138810	2.123105	C	4.325893	-0.574403	1.960984
C	-2.990134	-0.582022	1.995715	C	4.321069	-1.694287	1.157612
C	-3.010434	-1.764193	1.172381	C	5.544343	-2.125403	0.456374
C	-1.829640	-2.228868	0.554565	C	5.544873	-1.976271	-0.869378
C	-1.833180	-2.074028	-0.955082	C	4.325232	-1.396885	-1.457699
C	-3.012975	-1.510900	-1.526085	C	4.332155	-0.147856	-2.017350
C	-3.004262	-0.128378	-2.096491	C	5.553792	0.672223	-2.064963
C	-1.858954	0.644561	-2.158608	C	5.569127	1.755972	-1.285126
C	-1.815513	1.890209	-1.218782	Cl	-0.853513	-0.049817	-0.153961
C	-0.590757	2.214158	-0.519016	H	-6.317008	-0.341913	2.652077
C	-0.573007	2.067246	0.951324	H	-6.366187	-2.360113	1.193703
C	0.662034	1.510028	1.499550	H	-6.318486	2.271630	1.530675
C	0.651607	0.175232	2.022741	H	-6.340576	2.548019	-0.953267
C	-0.572940	-0.615703	2.066730	H	-6.345437	-2.088067	-1.612779
C	-0.585308	-1.845407	1.212629	H	-6.370628	0.204084	-2.616308
C	0.653397	-2.142253	0.524702	H	6.442790	2.073995	1.637909
C	0.643443	-2.016605	-0.918303	H	6.419633	-0.201235	2.633483
C	-0.577590	-1.738402	-1.597885	H	6.447983	2.380520	-1.183064
C	-0.613311	-0.146671	-1.866009	H	6.418042	0.361328	-2.638644
C	0.683008	0.616179	-1.939415	H	6.416075	-2.467052	1.000663
C	0.683039	1.811522	-1.175448	H	6.417383	-2.186405	-1.475310
C	1.909666	2.126506	-0.524269				

Table S7. XYZ coordinates of the N12-7 structure.

C	-3.661230	2.231208	-0.571184	C	1.280506	-1.599242	-1.446672
C	-2.450926	1.718642	-1.260774	C	-1.158028	-1.820029	-1.456205
C	-1.159527	2.193034	-0.655148	C	0.115531	-2.130321	-0.820700
C	-1.183196	2.109153	0.833227	C	-3.629329	-0.378268	-2.006001
C	-2.424404	1.579393	1.394879	C	-3.584709	-1.607981	-1.477537
C	-3.651820	2.119783	0.759327	C	-2.362259	-2.105577	-0.777392
C	0.057000	1.826406	-1.325383	C	-3.636088	-0.526345	1.956205
C	0.009425	1.618325	1.450664	C	3.744816	-1.640344	1.379797
C	1.234448	1.929331	0.796868	C	2.539614	-1.958345	0.605004
C	1.254773	2.048785	-0.611580	C	1.277238	-1.684804	1.272641
C	2.492266	1.516056	1.416396	C	0.103864	-2.209936	0.684181
C	2.484529	0.279585	1.997580	C	-1.160422	-1.807609	1.317145
C	1.244152	-0.472454	2.043487	C	-2.378228	-2.155713	0.650983
C	0.016576	0.253093	2.113831	C	-3.610232	-1.707140	1.320891

C	-1.189890	-0.495708	2.086543	H	4.623155	0.067527	-2.647466
C	-2.416217	0.296467	1.949291	H	4.613141	-2.158517	-1.507026
C	-2.454743	0.500903	-1.874493	H	4.590488	2.480769	-1.086458
C	-1.133477	-0.252350	-1.835113	H	4.568249	2.350857	1.401955
C	0.106731	0.518416	-2.204967	H	4.642923	-2.242219	1.300377
C	1.256130	-0.245343	-2.105796	H	4.571561	-0.165148	2.666457
C	2.531511	0.483795	-1.953207	H	-4.468942	-2.236208	-1.512827
C	2.524662	1.615092	-1.208424	H	-4.541200	-0.008519	-2.461230
C	3.707941	2.074167	0.803584	H	-4.514354	2.622427	-1.113227
C	3.725557	2.139833	-0.530516	H	-4.510301	2.410605	1.355936
C	3.711552	-0.524453	2.114923	H	-4.523319	-2.283878	1.218199
C	3.756309	-0.317361	-2.124054	H	-4.559456	-0.139673	2.371195
C	3.751522	-1.501334	-1.513682	Cl	-0.807946	-0.064987	-0.148714
C	2.525260	-1.843019	-0.764909				

Table S8. XYZ coordinates of the N12-14 structure.

C	-7.996800	2.153216	-0.201755	C	0.629654	-2.302462	0.262601
C	-7.994259	1.866094	1.101590	C	0.643118	-1.982838	-1.190803
C	-6.774591	1.221199	1.620637	C	-0.589133	-1.495582	-1.712122
C	-6.761611	-0.076096	2.030866	C	-0.538601	0.098051	-1.868090
C	-7.970826	-0.921883	1.997261	C	0.672229	0.943625	-2.146873
C	-7.971585	-1.903474	1.094637	C	0.623763	1.990630	-1.019879
C	-6.760116	-2.009119	0.259325	C	1.838301	2.091245	-0.270146
C	-6.757128	-1.713928	-1.065982	C	1.818610	1.748172	1.078780
C	-7.966544	-1.269324	-1.782768	C	3.076599	1.236552	1.620724
C	-7.968086	0.000327	-2.191530	C	3.071624	-0.108566	2.002062
C	-6.762543	0.784770	-1.866051	C	1.814544	-0.821409	1.839043
C	-6.778664	1.786353	-0.945754	C	1.832525	-1.875042	0.919565
C	-5.523958	2.072492	-0.262966	C	3.101910	-2.080095	0.229001
C	-5.519721	1.765176	1.118031	C	3.096447	-1.722796	-1.132197
C	-4.303419	1.321359	1.676995	C	1.836518	-1.276075	-1.687903
C	-4.293855	-0.085298	2.098529	C	1.827654	0.109684	-2.087897
C	-5.495837	-0.789449	1.925881	C	3.108610	0.799249	-1.887659
C	-5.495502	-1.847208	0.971458	C	3.107136	1.831070	-0.949397
C	-4.298201	-2.126980	0.302237	C	5.528752	-1.745264	-1.123919
C	-4.289268	-1.794411	-1.140275	C	4.334655	-1.279391	-1.684896
C	-5.491375	-1.260617	-1.635956	C	4.336445	0.119984	-2.101169
C	-5.493499	0.092646	-2.058381	C	5.532098	0.826643	-1.916949
C	-4.290268	0.810164	-1.928226	C	5.524413	1.873071	-0.933691
C	-4.311047	1.915775	-0.963253	C	6.773873	2.026480	-0.206376
C	-3.107862	2.136675	-0.259806	C	6.757174	1.710384	1.120506
C	-3.100184	1.796227	1.117081	C	7.963101	1.247423	1.830605
C	-1.824324	1.301478	1.633626	C	7.960688	-0.030023	2.217156
C	-1.818488	-0.054555	2.016728	C	6.752857	-0.808361	1.893990
C	-3.078414	-0.772781	1.875228	C	6.765560	-1.799795	0.955069
C	-3.079220	-1.851510	0.967736	C	7.991268	-2.154686	0.217303
C	-1.826653	-2.165968	0.286266	C	7.999875	-1.849281	-1.081577
C	-1.820446	-1.864474	-1.096693	C	6.784523	-1.203016	-1.607461
C	-3.069429	-1.267667	-1.611314	C	6.785628	0.104936	-2.003717

C	-3.068132	0.097886	-2.001082	C	8.001681	0.935884	-1.933641
C	-1.839716	0.844975	-1.814486	C	7.996649	1.908285	-1.019971
C	-1.844756	1.931660	-0.953800	H	8.873957	-1.994100	-1.704043
C	-0.584868	2.319332	-0.285316	H	8.856626	-2.564695	0.722653
C	-0.602486	1.946035	1.168716	H	8.871071	0.714274	-2.539809
C	0.578776	1.342813	1.685099	H	8.862482	2.530699	-0.831909
C	0.583066	-0.110998	2.096893	H	8.823057	-0.494323	2.679275
C	-0.602673	-0.860368	1.987225	H	8.824433	1.891022	1.958036
C	-0.592451	-2.014295	0.997530	H	-8.864757	2.560545	-0.704616
C	4.320817	2.120660	-0.268000	H	-8.860584	2.024024	1.731515
C	4.302212	1.781076	1.161390	H	-8.825890	-0.709264	2.626606
C	5.494856	1.262975	1.683009	H	-8.826837	-2.548208	0.935640
C	5.490297	-0.113494	2.084384	H	-8.821739	-1.921721	-1.906716
C	4.294856	-0.828049	1.936217	H	-8.825321	0.455794	-2.671140
C	4.312318	-1.911385	0.945903	Cl	-0.193370	-0.005788	-0.156870
C	5.524323	-2.098994	0.268061				

Table S9. XYZ coordinates of the N12-14+1aq structure.

C	7.906519	1.525394	1.626422	C	-3.191862	1.280992	-1.619318
C	7.914500	2.160184	0.453496	C	3.013631	-1.382850	1.731932
C	6.718688	1.958780	-0.387499	C	1.764794	-0.676206	1.988522
C	6.719985	1.262590	-1.541590	C	1.763388	0.770981	1.954625
C	7.921941	0.616208	-2.102654	C	0.535061	1.490293	1.618662
C	7.921741	-0.717111	-2.072955	C	0.527446	2.270910	0.315135
C	6.720678	-1.340404	-1.484606	C	-0.697196	2.196084	-0.480505
C	6.720061	-1.981102	-0.297889	C	-0.733528	1.520942	-1.764976
C	7.920416	-2.135320	0.545227	C	0.509985	0.736655	-1.858760
C	7.912635	-1.447729	1.687868	C	0.512780	-0.841532	-1.849967
C	6.705997	-0.633983	1.931773	C	-0.724661	-1.604678	-1.710744
C	6.705186	0.713602	1.905009	C	-0.689118	-2.213673	-0.376807
C	5.417326	1.340982	1.546022	C	0.527057	-2.227870	0.414404
C	5.428739	2.004868	0.328385	C	0.534037	-1.399239	1.681599
C	4.207904	2.161719	-0.421422	C	-0.640827	-0.688027	1.986801
C	4.204927	1.383848	-1.721243	C	-0.640843	0.799607	1.956251
C	5.433219	0.643116	-1.918866	C	-1.887050	1.369809	1.476365
C	5.432124	-0.738865	-1.882514	C	-1.919060	0.657203	-1.961573
C	4.208101	-1.466889	-1.644706	C	-1.913058	-0.745128	-1.928623
C	4.212574	-2.185135	-0.327582	C	-3.186249	-1.354929	-1.558277
C	5.431170	-1.998014	0.419324	C	-3.178960	-2.055270	-0.338144
C	5.420558	-1.283689	1.606281	C	-1.904474	-2.038818	0.375912
C	4.190643	-0.715378	2.103519	C	-1.881879	-1.281107	1.534699
C	4.189391	0.794998	2.060288	C	-3.140715	-0.650709	1.909966
C	3.014978	1.454939	1.659259	C	-3.143302	0.751469	1.878541
C	3.028060	2.247239	0.348897	C	-4.364940	1.404208	1.589412
C	1.790669	2.198560	-0.417710	C	-4.390674	2.120166	0.303273
C	1.807215	1.434603	-1.662195	C	-5.603560	2.053426	-0.397715
C	3.008916	0.747601	-2.115902	C	-5.614857	1.300523	-1.608396
C	3.010530	-0.832437	-2.041890	C	-4.417219	0.682906	-2.001409
C	1.812759	-1.498111	-1.583463	C	-4.413492	-0.779011	-1.966833

C	1.799799	-2.193968	-0.320859	C	-5.608906	-1.382109	-1.544217
C	3.027209	-2.209654	0.437063	C	-8.026986	-0.622450	2.153352
C	-5.594404	-2.073609	-0.298033	C	-8.029775	0.711213	2.121189
C	-4.381315	-2.102311	0.404691	H	-8.927978	-2.686437	0.020666
C	-4.358766	-1.322958	1.654605	H	-8.953356	-1.493054	-2.170683
C	-5.553855	-0.666938	1.983326	H	-8.879443	-1.197067	2.493484
C	-5.557145	0.758387	1.948639	H	-8.884771	1.297716	2.433524
C	-6.829450	1.346861	1.546728	H	-8.960451	1.365505	-2.238003
C	-6.852020	1.997717	0.352427	H	-8.939615	2.664363	-0.107718
C	-8.078256	2.109728	-0.458122	H	8.746547	1.548713	2.309237
C	-8.089132	1.415101	-1.597137	H	8.761010	2.740239	0.108033
C	-6.875328	0.630688	-1.889616	H	8.766829	1.194721	-2.454475
C	-6.872197	-0.731761	-1.856795	H	8.767240	-1.309793	-2.398900
C	-8.082596	-1.506910	-1.527301	H	8.772318	-2.718357	0.218793
C	-8.069216	-2.144956	-0.355720	H	8.757696	-1.427044	2.364316
C	-6.843894	-1.987635	0.448682	Cl	0.361497	0.000179	-0.225167
C	-6.824009	-1.279408	1.609424	O	3.260939	-0.083490	0.048265
C	-1.914389	2.066472	0.281368	H	4.164905	0.154889	-0.102728
C	-3.186770	2.041748	-0.435288	H	2.691702	0.557858	-0.331111

Table S10. XYZ coordinates of the N12-14+2aq structure.

C	7.928508	1.440238	1.719348	C	0.575635	-0.734077	-1.871065
C	7.947056	2.134692	0.580970	C	-0.706726	-1.457878	-1.725798
C	6.757531	1.979390	-0.278822	C	-0.678439	-2.190556	-0.451733
C	6.768223	1.334654	-1.463766	C	0.572434	-2.232120	0.299309
C	7.976407	0.714262	-2.038868	C	0.571613	-1.471562	1.609420
C	7.974645	-0.619081	-2.076285	C	-0.629483	-0.788656	1.961219
C	6.765883	-1.270728	-1.538356	C	-0.627392	0.690606	1.996005
C	6.754661	-1.972902	-0.385477	C	-1.885335	1.376243	1.680158
C	7.948691	-2.166585	0.459015	C	-1.905485	2.220109	0.436163
C	7.932220	-1.532570	1.632085	C	-3.108361	2.187911	-0.337224
C	6.723424	-0.730894	1.904735	C	-3.112188	1.464042	-1.684815
C	6.723523	0.616762	1.944889	C	-1.902306	0.854615	-2.112377
C	5.440299	1.267708	1.615170	C	-1.897251	-0.729411	-2.100005
C	5.463400	1.995650	0.428055	C	-3.112686	-1.382974	-1.737928
C	4.254473	2.177940	-0.322941	C	-3.107182	-2.163200	-0.444294
C	4.261517	1.468626	-1.658786	C	-1.899773	-2.214501	0.315469
C	5.487870	0.736554	-1.886622	C	-1.881355	-1.462803	1.620321
C	5.484529	-0.647788	-1.919291	C	-3.072342	-0.822190	2.042614
C	4.261171	-1.386683	-1.726155	C	-3.072608	0.709256	2.069488
C	4.254213	-2.161153	-0.445040	C	3.057057	-2.227367	0.312801
C	5.462488	-2.029210	0.319400	C	3.037229	-1.466364	1.638399
C	5.441405	-1.366857	1.543072	C	-0.675809	2.234651	-0.338353
C	4.214258	-0.823645	2.055543	C	-0.704777	1.561095	-1.648374
C	4.213342	0.696900	2.087219	C	0.577913	0.837036	-1.824898
C	3.039674	1.372570	1.709457	C	-5.584659	1.331287	-1.483619
C	3.061521	2.229594	0.447145	C	-4.329675	0.745155	-1.939184
C	1.832475	2.238090	-0.324515	C	-4.324314	-0.656713	-1.961156
C	1.864693	1.555295	-1.637752	C	-5.581794	-1.272472	-1.552196

C	3.059190	0.865144	-2.099458	C	-6.849496	-1.999882	0.351630
C	-5.571300	-1.974207	-0.391178	C	0.572084	1.396078	1.696285
C	-4.304123	-2.067963	0.320239	C	0.575686	2.236340	0.422665
C	-4.283593	-1.387889	1.557338	C	-6.834511	-1.353504	1.534008
C	-5.543137	-0.727425	1.889523	C	-8.020554	-0.722072	2.145859
C	-5.544560	0.626235	1.919293	C	-8.021889	0.613019	2.177248
C	-6.836010	1.274935	1.599583	H	-8.900474	-2.745326	-0.160725
C	-6.851922	1.972980	0.446794	H	-8.917332	-1.452262	-2.293967
C	-8.057365	2.164919	-0.382971	H	-8.862236	-1.310850	2.488718
C	-8.066651	1.524195	-1.554576	H	-8.865977	1.182424	2.546079
C	-6.871589	0.713548	-1.860911	H	-8.923727	1.540404	-2.216178
C	-6.869026	-0.634726	-1.892885	H	-8.905716	2.736723	-0.028236
C	-8.062700	-1.462750	-1.629139	H	8.761648	1.428737	2.410933
C	-8.053458	-2.155140	-0.487510	H	8.796741	2.731679	0.273952
H	4.213001	0.124811	-0.092100	H	8.829182	1.307377	-2.344345
H	2.782464	0.613431	-0.341130	H	8.826822	-1.195186	-2.414453
C	-4.284572	1.300824	1.620439	H	8.802701	-2.735582	0.113624
C	-4.306376	2.039096	0.416156	H	8.771431	-1.544845	2.316081
C	-5.576375	1.991592	-0.299551	Cl	0.572283	0.011426	-0.253751
C	3.056391	-0.739935	-2.101514	O	-2.142978	-0.075429	0.014874
C	1.866007	-1.449089	-1.707357	H	-3.055739	0.147555	-0.118538
C	1.836627	-2.191723	-0.447638	H	-1.610335	0.592012	-0.372074
C	1.784235	-0.784418	1.957618	O	3.293730	-0.081163	0.023280
C	1.782729	0.681397	1.995053				

Table S11. XYZ coordinates of the N20-10 structure.

C	2.800786	2.414055	-3.364589	C	3.225249	-2.375024	-3.452145
C	3.138640	3.379825	-2.322812	C	4.462441	-1.922116	-3.993980
C	4.472563	3.720641	-2.000908	C	4.617176	-0.637907	-4.409626
C	4.854015	3.921815	-0.705051	C	-4.616645	-0.638513	4.409481
C	0.416180	2.661141	-2.765008	C	-4.461913	-1.922669	3.993680
C	0.774730	3.453032	-1.653700	C	-3.224720	-2.375488	3.451778
C	2.165078	3.641722	-1.352486	C	-3.160308	-3.305941	2.335990
C	2.560177	3.785441	0.005543	C	-1.901340	-3.495983	1.693236
C	3.924761	3.748235	0.350862	C	-1.848337	-3.768610	0.314742
C	4.306117	3.166684	1.610236	C	-0.592505	-3.757321	-0.365895
C	5.664623	2.845458	1.926699	C	-0.547870	-3.341615	-1.724700
C	5.968949	1.743684	2.652802	C	0.701401	-3.062966	-2.322928
C	-1.934469	2.897377	-2.169378	C	0.794462	-2.046766	-3.288287
C	-1.565831	3.583166	-1.006145	C	2.089782	-1.601293	-3.708428
C	-0.195838	3.729539	-0.685887	C	2.250994	-0.236616	-4.080582
C	0.196318	3.729501	0.686396	C	3.545723	0.291541	-4.262930
C	1.566325	3.583098	1.006625	C	3.783474	1.642067	-3.965298
C	1.934982	2.897127	2.169768	C	-4.308689	-3.667999	1.638333
C	3.304553	2.550659	2.370694	C	-4.279767	-3.798065	0.246515
C	3.607964	1.351347	3.078267	C	-3.044320	-3.705316	-0.430974
C	4.929454	0.851158	3.069695	C	-2.999349	-3.153453	-1.744092
C	5.140385	-0.526130	3.140005	C	-1.722088	-2.826554	-2.304102
C	-5.968425	1.743996	-2.652571	C	-1.617808	-1.733144	-3.177828

C	-5.664102	2.845667	-1.926315	C	-0.338687	-1.256391	-3.546616
C	-4.305591	3.166827	-1.609805	C	-0.154156	0.135173	-3.775830
C	-3.924243	3.748232	-0.350369	C	1.170426	0.651705	-3.876257
C	-2.559667	3.785394	-0.005045	C	1.450100	1.958902	-3.441873
C	-2.164579	3.641526	1.352952	C	-5.467561	-3.680506	-0.539943
C	-0.774238	3.452873	1.654181	C	-5.411740	-3.099279	-1.764021
C	-0.415682	2.660775	2.765336	C	-4.171616	-2.642950	-2.305708
C	0.925199	2.261424	2.927944	C	-4.064276	-1.409408	-3.063136
C	1.216164	0.997489	3.503934	C	-2.762098	-0.902088	-3.342442
C	2.548965	0.486000	3.423525	C	-2.548442	0.486449	-3.423477
C	2.762633	-0.902544	3.342292	C	-1.215666	0.997976	-3.503865
C	4.064823	-1.409824	3.062918	C	-0.924701	2.261805	-2.927662
C	4.172168	-2.643303	2.305366	C	-5.139842	-0.525723	-3.140091
C	5.412289	-3.099547	1.763592	C	-4.928922	0.851552	-3.069604
C	5.468110	-3.680611	0.539436	C	-3.607433	1.351727	-3.078083
C	-4.853502	3.921668	0.705554	C	-3.304026	2.550923	-2.370342
C	-4.472057	3.720333	2.001385	H	5.593533	-0.278153	-4.712936
C	-3.138132	3.379478	2.323231	H	5.325097	-2.575881	-3.976477
C	-2.800261	2.413576	3.364861	H	5.280709	-3.575081	-2.106887
C	-1.449584	1.958416	3.442071	H	6.425687	-3.922803	0.093305
C	-1.169924	0.651159	3.876222	H	6.334899	-2.888535	2.289190
C	0.154643	0.134627	3.775692	H	6.147958	-0.882971	2.970322
C	0.339202	-1.256933	3.546459	H	7.003153	1.453848	2.798235
C	1.618328	-1.733592	3.177565	H	6.466077	3.434985	1.500323
C	1.722625	-2.826913	2.303705	H	5.905812	4.052143	-0.487858
C	2.999902	-3.153752	1.743674	H	5.234623	3.700915	-2.769629
C	3.044868	-3.705445	0.430463	H	4.815438	1.968386	-3.949259
C	4.280317	-3.798056	-0.247056	H	-6.334342	-2.888171	-2.289592
C	4.309235	-3.667799	-1.638858	H	-6.425137	-3.922750	-0.093837
C	-3.782941	1.641499	3.965448	H	-5.280167	-3.575346	2.106369
C	-3.545189	0.290942	4.262903	H	-5.324565	-2.576437	3.976079
C	-2.250470	-0.237184	4.080457	H	-5.593001	-0.278790	4.712830
C	-2.089258	-1.601803	3.708156	H	-4.814903	1.967828	3.949458
C	-0.793946	-2.047264	3.288001	H	-5.234126	3.700487	2.770092
C	-0.700882	-3.063333	2.322522	H	-5.905301	4.052009	0.488379
C	0.548385	-3.341867	1.724226	H	-6.465547	3.435138	-1.499844
C	0.593023	-3.757434	0.365376	H	-7.002627	1.454170	-2.798043
C	1.848865	-3.768625	-0.315259	H	-6.147415	-0.882590	-2.970457
C	1.901871	-3.495748	-1.693727	Cl	-0.009751	0.005129	0.000502
C	3.160847	-3.305615	-2.336460				

Table S12. XYZ coordinates of the G54 structure.

C	-1.930829	3.254055	-0.107099	C	-2.772539	-5.541116	-0.057788
C	-0.604808	2.781978	-0.150396	C	-4.373596	-3.652876	-0.030058
C	-0.345090	1.381094	-0.173747	C	-5.985140	-1.773941	0.010894
C	-1.416038	0.469869	-0.138516	C	-6.230241	-0.447566	0.015881
C	-2.758154	0.946324	-0.094005	C	-5.395765	1.883177	-0.008748
C	-3.014464	2.331433	-0.081442	C	-4.573684	4.218544	-0.020712
C	-1.160521	-0.911976	-0.146567	C	-3.546530	5.092760	-0.042520

C	0.164454	-1.380727	-0.190249	C	-1.111321	5.534507	-0.102880
C	1.231695	-0.470263	-0.223904	C	1.320917	5.990888	-0.149492
C	0.976663	0.910944	-0.216958	C	2.590522	5.537171	-0.178712
C	2.572710	-0.946265	-0.247097	C	4.188549	3.648940	-0.228927
C	2.828537	-2.327417	-0.235448	C	5.798814	1.772076	-0.260433
C	1.748113	-3.251997	-0.199780	C	6.042668	0.446139	-0.268473
C	0.422011	-2.781626	-0.176954	C	5.208595	-1.881642	-0.258874
C	-0.647771	-3.695796	-0.136878	C	4.389447	-4.215025	-0.224670
C	-1.989355	-3.221244	-0.104876	C	3.365369	-5.091838	-0.198342
C	-2.244586	-1.836048	-0.106770	C	0.931046	-5.533501	-0.148114
C	3.641869	-0.034424	-0.261629	H	-1.297152	-7.055444	-0.074256
C	3.384392	1.363310	-0.249814	H	-3.600494	-6.240333	-0.025193
C	2.059620	1.834224	-0.227561	H	-5.198525	-4.357200	0.001990
C	1.805855	3.216784	-0.202787	H	-6.805677	-2.482063	0.040515
C	0.466240	3.695219	-0.166218	H	-7.249517	-0.079465	0.049460
C	-3.830256	0.033434	-0.061275	H	-6.417730	2.246365	0.026571
C	-3.571715	-1.366065	-0.067131	H	-5.596672	4.576009	0.014240
C	2.885992	4.132705	-0.205633	H	-3.736496	6.160115	-0.025070
C	4.458214	2.284133	-0.249225	H	-1.307417	6.601622	-0.081600
C	4.163830	-2.797692	-0.242625	H	1.119060	7.055855	-0.125854
C	4.973286	-0.510667	-0.266967	H	3.420616	6.234757	-0.177996
C	2.000937	-4.645309	-0.183252	H	5.015131	4.352044	-0.222873
C	-0.386110	-5.087516	-0.124868	H	6.619170	2.480920	-0.252932
C	-3.069425	-4.136619	-0.064966	H	7.061867	0.076312	-0.266721
C	-4.645697	-2.289061	-0.029655	H	6.231521	-2.243812	-0.255141
C	-5.162953	0.512095	-0.018793	H	5.413860	-4.570343	-0.226769
C	-4.350067	2.800707	-0.037648	H	3.556532	-6.158888	-0.180270
C	-2.182737	4.647471	-0.085485	H	1.129014	-6.600381	-0.133105
C	0.205157	5.087254	-0.140492	Cl	1.852484	0.004300	2.884541
C	-1.500993	-5.990548	-0.085021				

Table S13. XYZ coordinates of the B19N19 structure.

B	-2.970417	0.666923	-1.105862	B	-0.282319	1.572691	2.186047
N	-3.179364	-0.778963	-1.212947	N	-0.360035	-1.913800	2.200798
N	-1.990078	0.861836	-2.167345	N	0.656588	-2.676686	-0.150390
N	-3.051426	1.421634	0.097302	N	1.710349	-1.357863	-2.139989
B	-2.798736	-1.392666	0.020608	N	2.955432	0.788404	-1.527697
B	-1.940900	-0.614139	-2.018969	N	1.737804	2.441455	-0.701908
B	-0.755311	1.513192	-1.764720	N	0.720569	2.434033	1.540501
B	-1.804020	2.128006	0.373329	N	0.641741	0.573096	2.811805
B	-2.589390	0.655722	1.293152	B	0.643509	-0.843874	2.516567
N	-2.707071	-0.781462	1.378062	B	0.739097	-2.290086	1.274804
N	-1.802544	-2.433790	0.142248	B	1.754843	-2.076718	-0.847922
N	-0.770749	-1.446427	-2.032278	B	2.759387	-0.609816	-1.452058
N	0.469856	0.903703	-2.373656	B	2.667929	1.407101	-0.223535
N	-0.766715	2.498190	-0.589767	B	1.566348	1.280018	1.880838
N	-1.656703	1.685868	1.799082	N	1.815266	-1.518865	1.915016
B	-1.564089	-1.739316	1.447578	N	2.960579	-1.437759	-0.267075
B	-0.656681	-2.274351	-0.748975	N	2.657358	0.738006	1.076196

B 0.457172 -0.628820 -2.293695	B 2.617071 -0.754664 0.977347
B 1.668615 1.465368 -1.799226	Cl -0.033061 -0.017562 0.140046
B 0.543098 2.594285 0.091395	

Table S14. XYZ coordinates of the Au20 structure.

Au 2.603382 1.222393 1.620768	Au -1.805598 2.745813 0.289204
Au 3.597457 -0.432141 -3.390286	Au -1.026907 -1.102183 -2.281273
Au -1.984226 0.237503 1.867506	Au -1.858014 -4.485975 1.021504
Au 1.983958 -1.606670 0.977005	Au 1.863396 2.001182 4.139477
Au -0.028410 2.450094 2.208742	Au -3.166377 0.374213 -0.848471
Au -0.603451 -2.364125 2.219411	Au 1.806877 -1.939085 -1.967232
Au 3.162228 0.432475 -0.827011	Au -3.601598 2.916276 -1.772672
Au 0.033673 -3.252911 -0.531047	Au -1.171965 1.856014 -2.462798
Au 0.604943 -0.256013 3.229873	Au 1.165997 0.764329 -2.988292
Au -2.598952 -2.031557 0.058775	Cl -0.000526 0.000657 -0.001023
Au 1.023701 2.470227 -0.562962	

Table S15. F30 isotopic frequencies and shifts (in cm^{-1}).

ν^{35}Cl	ν^{37}Cl	Isotopic shift	ν^{35}Cl	ν^{37}Cl	Isotopic shift
90.9203	90.8087	0.1	831.9426	831.9426	0.0
315.4924	312.8903	2.6	837.9158	837.3504	0.6
341.1561	334.2320	6.9	851.9845	851.9406	0.0
419.5169	419.5168	0.0	928.2779	928.2140	0.1
433.6739	428.5418	5.1	943.7684	943.7031	0.1
439.4162	430.8480	8.6	964.8291	964.8263	0.0
442.8774	442.8740	0.0	976.6230	976.6230	0.0
445.4038	445.2578	0.1	985.1283	985.1283	0.0
456.3582	456.2340	0.1	1014.0653	1014.0648	0.0
470.0167	469.9375	0.1	1034.0192	1034.0192	0.0
476.3036	474.9570	1.3	1047.2939	1047.2939	0.0
476.5273	476.3040	0.2	1084.8988	1084.8963	0.0
489.3129	489.0254	0.3	1097.1046	1097.0621	0.0
537.9142	537.8619	0.1	1120.7788	1120.7787	0.0
542.1344	541.9961	0.1	1126.3357	1126.3355	0.0
550.3182	550.1857	0.1	1126.9889	1126.9851	0.0
560.4408	560.1287	0.3	1152.2059	1152.1945	0.0
567.1373	567.1373	0.0	1153.3902	1153.3313	0.1
576.3089	576.1992	0.1	1158.9316	1158.9283	0.0
583.7314	583.5893	0.1	1182.6786	1182.6786	0.0
607.7558	607.4876	0.3	1205.1681	1205.1681	0.0
622.8662	622.1283	0.7	1213.8548	1213.8481	0.0
633.2901	633.2901	0.0	1239.3310	1239.3310	0.0
648.1622	648.1567	0.0	1241.3377	1241.3376	0.0
653.3721	653.1822	0.2	1249.1550	1249.1365	0.0
654.6822	654.6822	0.0	1265.5751	1265.5748	0.0
664.9044	664.9038	0.0	1278.9489	1278.9489	0.0
676.0934	676.0885	0.0	1296.0950	1296.0946	0.0
684.2942	684.2807	0.0	1313.2623	1313.2623	0.0

$\nu^{35}Cl$	$\nu^{37}Cl$	Isotopic shift	$\nu^{35}Cl$	$\nu^{37}Cl$	Isotopic shift
684.6171	684.5983	0.0	1324.8359	1324.8329	0.0
694.3151	694.3151	0.0	1326.3908	1326.3907	0.0
700.7956	700.7912	0.0	1333.8989	1333.8910	0.0
703.7482	703.7482	0.0	1360.6069	1360.6068	0.0
710.3803	710.3656	0.0	1395.1659	1395.1658	0.0
711.6691	711.0187	0.7	1398.1907	1398.1862	0.0
723.5935	723.5864	0.0	1413.7568	1413.7568	0.0
730.6602	730.6592	0.0	1414.0853	1414.0742	0.0
731.5571	731.5530	0.0	1416.5458	1416.5455	0.0
746.5274	746.4501	0.1	1442.7918	1442.7913	0.0
747.4585	747.4585	0.0	1448.1942	1448.1864	0.0
760.9495	759.4666	1.5	1448.8059	1448.8020	0.0
773.3138	771.9062	1.4	1468.3170	1468.3155	0.0
779.3048	779.3047	0.0	1515.2137	1515.2131	0.0
804.0118	803.9889	0.0			

Table S16. N16-9 isotopic frequencies and shifts (in cm^{-1}).

$\nu^{35}Cl$	$\nu^{37}Cl$	Isotopic shift	$\nu^{35}Cl$	$\nu^{37}Cl$	Isotopic shift
63.8974	62.2448	1.7	948.5084	948.5069	0.0
83.8302	83.8302	0.0	948.5217	948.5202	0.0
99.9394	99.9394	0.0	965.4867	965.4865	0.0
114.0631	114.0631	0.0	985.0841	985.0841	0.0
126.6432	126.6432	0.0	1004.4491	1004.4491	0.0
181.7322	181.3712	0.4	1007.3150	1007.3150	0.0
181.7526	181.3904	0.4	1007.3201	1007.3201	0.0
201.3722	201.3716	0.0	1009.2789	1009.2789	0.0
211.0978	211.0978	0.0	1009.2878	1009.2877	0.0
216.1644	216.1644	0.0	1010.9798	1010.9798	0.0
216.1792	216.1792	0.0	1011.7927	1011.7927	0.0
228.3150	228.3150	0.0	1014.5294	1014.5294	0.0
238.8588	238.3766	0.5	1016.4426	1016.4426	0.0
238.8732	238.3954	0.5	1016.4563	1016.4563	0.0
250.9396	250.9324	0.0	1028.1054	1028.1046	0.0
254.7681	254.7681	0.0	1028.5717	1028.5717	0.0
255.9629	255.9629	0.0	1051.9475	1051.9475	0.0
255.9789	255.9789	0.0	1061.5100	1061.5100	0.0
267.6390	267.6390	0.0	1061.5305	1061.5305	0.0
296.2218	290.5498	5.7	1104.2475	1104.2475	0.0
296.5391	290.8621	5.7	1106.3034	1106.3034	0.0
312.3263	312.3263	0.0	1106.3306	1106.3306	0.0
318.8113	318.8112	0.0	1107.8003	1107.8003	0.0
318.8427	318.8424	0.0	1107.8401	1107.8401	0.0
322.2116	322.1057	0.1	1110.3936	1110.3936	0.0
322.3089	322.2034	0.1	1155.0811	1155.0811	0.0
323.0443	322.9694	0.1	1164.5646	1164.5646	0.0
332.7009	332.7009	0.0	1166.2770	1166.2770	0.0
357.3950	357.3950	0.0	1170.6603	1170.6603	0.0
358.5790	358.5790	0.0	1170.6728	1170.6728	0.0

$\nu^{35}Cl$	$\nu^{37}Cl$	Isotopic shift	$\nu^{35}Cl$	$\nu^{37}Cl$	Isotopic shift
376.4983	376.3702	0.1	1178.0948	1178.0948	0.0
376.5518	376.4221	0.1	1181.7144	1181.7144	0.0
395.2125	395.2125	0.0	1182.8321	1182.8321	0.0
397.4183	397.4183	0.0	1183.0413	1183.0413	0.0
415.8398	415.8398	0.0	1183.0637	1183.0637	0.0
415.8684	415.8684	0.0	1232.4565	1232.4565	0.0
419.7903	419.7902	0.0	1235.5189	1235.5189	0.0
422.5043	421.5877	0.9	1235.5550	1235.5550	0.0
422.5703	421.6502	0.9	1249.2317	1249.2317	0.0
431.0269	431.0269	0.0	1250.8070	1250.8065	0.0
431.3863	431.3863	0.0	1250.8127	1250.8123	0.0
434.6339	434.5354	0.1	1255.5598	1255.5596	0.0
440.0567	440.0566	0.0	1286.8332	1286.8332	0.0
443.1113	443.1113	0.0	1286.8793	1286.8793	0.0
444.5100	444.5100	0.0	1287.8632	1287.8632	0.0
474.7580	474.7580	0.0	1291.1641	1291.1641	0.0
474.7987	474.7987	0.0	1293.2811	1293.2811	0.0
485.7366	485.7366	0.0	1293.2977	1293.2977	0.0
488.5346	488.4743	0.1	1303.4754	1303.4754	0.0
488.6022	488.5415	0.1	1309.9376	1309.9376	0.0
504.8441	504.8441	0.0	1324.7239	1324.7239	0.0
512.4339	512.4339	0.0	1324.7633	1324.7633	0.0
512.4707	512.4707	0.0	1326.4881	1326.4881	0.0
522.8081	522.8081	0.0	1336.6752	1336.6752	0.0
527.2425	527.2157	0.0	1336.6893	1336.6891	0.0
527.2576	527.2309	0.0	1336.6981	1336.6979	0.0
529.1559	529.1559	0.0	1349.8104	1349.8104	0.0
529.1661	529.1661	0.0	1351.0076	1351.0073	0.0
537.5952	537.5952	0.0	1351.2975	1351.2975	0.0
543.7598	543.7598	0.0	1354.5385	1354.5385	0.0
545.1779	545.1779	0.0	1354.8533	1354.8533	0.0
560.0766	560.0766	0.0	1357.2598	1357.2597	0.0
562.8390	562.8390	0.0	1357.2927	1357.2926	0.0
576.0985	576.0985	0.0	1373.8410	1373.8410	0.0
576.1147	576.1146	0.0	1373.8736	1373.8736	0.0
576.5203	576.4149	0.1	1381.0059	1381.0059	0.0
576.5655	576.4612	0.1	1400.1353	1400.1353	0.0
579.9724	579.9723	0.0	1401.2649	1401.2649	0.0
593.9953	593.9292	0.1	1402.3625	1402.3606	0.0
594.1286	594.0612	0.1	1405.7512	1405.7512	0.0
599.2833	599.2833	0.0	1405.7631	1405.7631	0.0
602.1980	602.1980	0.0	1425.4927	1425.4925	0.0
615.5755	615.5755	0.0	1425.5132	1425.5130	0.0
617.1323	617.1323	0.0	1428.4130	1428.4130	0.0
634.9674	634.9674	0.0	1434.9731	1434.9731	0.0
635.4221	635.4221	0.0	1437.6665	1437.6665	0.0
635.4416	635.4416	0.0	1437.6737	1437.6737	0.0
639.4800	639.4768	0.0	1453.8745	1453.8745	0.0
639.4919	639.4887	0.0	1453.8985	1453.8985	0.0
643.4144	643.4144	0.0	1456.4932	1456.4932	0.0

ν^{35}Cl	ν^{37}Cl	Isotopic shift	ν^{35}Cl	ν^{37}Cl	Isotopic shift
646.1045	646.1045	0.0	1457.9197	1457.9197	0.0
656.3225	656.3181	0.0	1462.5027	1462.5027	0.0
656.3529	656.3484	0.0	1474.1757	1474.1755	0.0
666.6809	666.6809	0.0	1474.1908	1474.1906	0.0
666.7124	666.7124	0.0	1479.7872	1479.7872	0.0
667.2512	667.2512	0.0	1485.5980	1485.5980	0.0
684.7470	684.7470	0.0	1507.7247	1507.7244	0.0
693.1072	693.1072	0.0	1512.0269	1512.0269	0.0
697.1367	697.1367	0.0	1512.0423	1512.0423	0.0
699.2803	699.2766	0.0	1512.2060	1512.2060	0.0
699.3173	699.3138	0.0	1512.9968	1512.9968	0.0
706.2422	706.2421	0.0	1530.4193	1530.4193	0.0
719.7386	719.7386	0.0	1530.4507	1530.4507	0.0
719.7827	719.7827	0.0	1532.4061	1532.4061	0.0
723.6491	723.6491	0.0	1567.2275	1567.2275	0.0
730.0397	730.0397	0.0	1569.7388	1569.7387	0.0
732.6655	732.6655	0.0	1569.7725	1569.7725	0.0
737.6782	737.6782	0.0	1578.5108	1578.5108	0.0
756.1359	756.1359	0.0	1597.2123	1597.2123	0.0
763.1876	763.1766	0.0	1599.2210	1599.2210	0.0
763.1988	763.1878	0.0	1599.2467	1599.2467	0.0
781.5954	781.5954	0.0	1599.2826	1599.2826	0.0
781.6320	781.6320	0.0	1608.7955	1608.7954	0.0
782.8935	782.8935	0.0	1608.8274	1608.8273	0.0
786.3834	786.3834	0.0	1631.4994	1631.4994	0.0
786.4759	786.4759	0.0	1641.1020	1641.1020	0.0
791.6957	791.6956	0.0	1644.0709	1644.0709	0.0
796.6754	796.6754	0.0	1664.9113	1664.9113	0.0
796.6985	796.6985	0.0	1667.2777	1667.2777	0.0
797.0760	797.0760	0.0	1667.2933	1667.2932	0.0
800.3200	800.3200	0.0	1668.5149	1668.5149	0.0
800.9711	800.9711	0.0	1668.5273	1668.5273	0.0
802.3644	802.3644	0.0	1668.5503	1668.5503	0.0
802.8417	802.8383	0.0	1670.1251	1670.1251	0.0
802.8626	802.8591	0.0	3204.0058	3204.0058	0.0
808.3628	808.3628	0.0	3204.0524	3204.0524	0.0
809.1375	809.1375	0.0	3206.0633	3206.0633	0.0
823.4191	823.4183	0.0	3206.0643	3206.0643	0.0
823.4531	823.4524	0.0	3206.0985	3206.0985	0.0
830.8620	830.8620	0.0	3206.0997	3206.0997	0.0
830.8867	830.8867	0.0	3208.5422	3208.5422	0.0
839.3576	839.3576	0.0	3208.5782	3208.5782	0.0
852.1411	852.1410	0.0	3221.6667	3221.6667	0.0
852.1727	852.1727	0.0	3221.6742	3221.6742	0.0
853.3696	853.3696	0.0	3223.8743	3223.8743	0.0
853.3820	853.3820	0.0	3223.8776	3223.8776	0.0
861.4298	861.4298	0.0	3223.8838	3223.8838	0.0
870.6798	870.6798	0.0	3223.8902	3223.8902	0.0
888.9194	888.9194	0.0	3225.5255	3225.5255	0.0
903.1421	903.1421	0.0	3225.7582	3225.7582	0.0

ν^{35}Cl	ν^{37}Cl	Isotopic shift	ν^{35}Cl	ν^{37}Cl	Isotopic shift
912.4991	912.4991	0.0			

Table S17. G54 isotopic frequencies and shifts (in cm^{-1}).

ν^{35}Cl	ν^{37}Cl	Isotopic shift	ν^{35}Cl	ν^{37}Cl	Isotopic shift
11.2997	11.0137	0.3	975.5477	975.5477	0.0
21.7136	21.1740	0.5	977.1378	977.1378	0.0
39.8157	39.8100	0.0	979.1439	979.1439	0.0
39.9815	39.9687	0.0	979.7104	979.7104	0.0
51.3300	51.1412	0.2	980.4660	980.4660	0.0
76.7907	76.7887	0.0	1015.2132	1015.2132	0.0
101.9279	101.3726	0.6	1015.4459	1015.4459	0.0
104.0344	102.7705	1.3	1024.1728	1024.1728	0.0
131.7738	131.7727	0.0	1024.4370	1024.4370	0.0
140.8667	140.2858	0.6	1034.7352	1034.7352	0.0
146.8235	146.8110	0.0	1059.8618	1059.8618	0.0
146.9496	146.9423	0.0	1060.1491	1060.1491	0.0
219.4848	219.4842	0.0	1110.7052	1110.7051	0.0
220.0345	220.0267	0.0	1119.2581	1119.2581	0.0
237.3227	237.3108	0.0	1135.3374	1135.3374	0.0
238.2465	238.2462	0.0	1152.3957	1152.3957	0.0
239.1275	239.1195	0.0	1152.4724	1152.4724	0.0
256.7920	256.7919	0.0	1165.5226	1165.5226	0.0
256.9487	256.9486	0.0	1188.0966	1188.0966	0.0
273.0116	273.0115	0.0	1188.2333	1188.2333	0.0
273.1207	273.1207	0.0	1195.0233	1195.0233	0.0
293.9555	293.9544	0.0	1195.3751	1195.3751	0.0
300.6969	300.6961	0.0	1196.3091	1196.3091	0.0
312.2673	312.2673	0.0	1196.5207	1196.5207	0.0
335.8104	335.8103	0.0	1217.9027	1217.9027	0.0
349.6211	349.6211	0.0	1218.3844	1218.3844	0.0
356.0686	356.0684	0.0	1234.6669	1234.6669	0.0
356.6318	356.6301	0.0	1244.3036	1244.3036	0.0
382.4531	382.4531	0.0	1263.7453	1263.7453	0.0
382.6145	382.6145	0.0	1285.3925	1285.3924	0.0
388.1932	388.1928	0.0	1299.3501	1299.3500	0.0
395.6955	395.6951	0.0	1308.4048	1308.4048	0.0
396.7889	396.7879	0.0	1308.4719	1308.4719	0.0
399.5714	399.5714	0.0	1312.0949	1312.0949	0.0
404.5684	404.5682	0.0	1315.2466	1315.2466	0.0
405.2093	405.2092	0.0	1315.8718	1315.8718	0.0
461.3816	461.3816	0.0	1343.1477	1343.1477	0.0
462.7153	462.7153	0.0	1343.7859	1343.7858	0.0
480.4633	480.4633	0.0	1344.2142	1344.2142	0.0
480.4984	480.4983	0.0	1344.5980	1344.5980	0.0
483.5331	483.5331	0.0	1368.4553	1368.4553	0.0
496.9764	496.9764	0.0	1382.6414	1382.6414	0.0
504.7127	504.7127	0.0	1383.9221	1383.9221	0.0
513.4990	513.4990	0.0	1390.5546	1390.5546	0.0

$\nu^{35}Cl$	$\nu^{37}Cl$	Isotopic shift	$\nu^{35}Cl$	$\nu^{37}Cl$	Isotopic shift
513.6106	513.6106	0.0	1416.6745	1416.6745	0.0
542.6462	542.6462	0.0	1416.9099	1416.9099	0.0
544.6527	544.6527	0.0	1431.6066	1431.6066	0.0
559.4545	559.4545	0.0	1432.0917	1432.0917	0.0
560.2062	560.2062	0.0	1442.1141	1442.1141	0.0
581.1333	581.1332	0.0	1442.9301	1442.9301	0.0
581.2681	581.2680	0.0	1443.0647	1443.0647	0.0
589.9014	589.9011	0.0	1453.8138	1453.8138	0.0
591.9634	591.9634	0.0	1455.9854	1455.9854	0.0
593.5616	593.5616	0.0	1460.6901	1460.6901	0.0
607.6662	607.6662	0.0	1460.9165	1460.9165	0.0
608.0787	608.0787	0.0	1464.6828	1464.6828	0.0
608.4341	608.4341	0.0	1464.7188	1464.7188	0.0
608.4933	608.4933	0.0	1469.5575	1469.5575	0.0
637.9898	637.9898	0.0	1471.0073	1471.0073	0.0
638.3039	638.3039	0.0	1471.7467	1471.7466	0.0
643.4933	643.4933	0.0	1483.9236	1483.9236	0.0
645.3855	645.3855	0.0	1512.7922	1512.7922	0.0
647.5442	647.5442	0.0	1522.3005	1522.3005	0.0
648.6851	648.6851	0.0	1522.4500	1522.4500	0.0
649.0082	649.0081	0.0	1533.7716	1533.7716	0.0
649.4311	649.4311	0.0	1536.7098	1536.7098	0.0
666.4273	666.4272	0.0	1538.9444	1538.9444	0.0
666.5334	666.5333	0.0	1540.9755	1540.9755	0.0
669.4649	669.4649	0.0	1545.2544	1545.2544	0.0
669.5900	669.5900	0.0	1570.2460	1570.2460	0.0
735.3451	735.3451	0.0	1570.7469	1570.7469	0.0
740.1304	740.1304	0.0	1577.0639	1577.0639	0.0
741.9535	741.9535	0.0	1577.3857	1577.3857	0.0
759.9395	759.9392	0.0	1601.4780	1601.4780	0.0
766.4441	766.4440	0.0	1638.5595	1638.5595	0.0
772.6515	772.6515	0.0	1638.8070	1638.8070	0.0
776.1379	776.1378	0.0	1644.7641	1644.7641	0.0
779.6835	779.6835	0.0	1658.1596	1658.1596	0.0
779.8516	779.8516	0.0	1658.9883	1658.9883	0.0
809.5131	809.5130	0.0	1668.6217	1668.6217	0.0
811.1573	811.1573	0.0	1679.5418	1679.5418	0.0
812.5950	812.5950	0.0	1679.5888	1679.5888	0.0
822.5376	822.5376	0.0	1688.8184	1688.8184	0.0
826.9540	826.9540	0.0	1702.1601	1702.1601	0.0
827.8796	827.8796	0.0	1704.1567	1704.1567	0.0
835.9646	835.9646	0.0	1708.2661	1708.2661	0.0
836.6545	836.6544	0.0	1708.5654	1708.5654	0.0
837.1043	837.1043	0.0	1710.7264	1710.7264	0.0
854.4506	854.4504	0.0	3174.4613	3174.4613	0.0
859.1618	859.1618	0.0	3174.9866	3174.9866	0.0
859.2556	859.2556	0.0	3175.2910	3175.2910	0.0
860.0954	860.0954	0.0	3175.6014	3175.6014	0.0
861.6316	861.6316	0.0	3175.9206	3175.9206	0.0
861.7461	861.7461	0.0	3176.5468	3176.5468	0.0

$\nu^{35}Cl$	$\nu^{37}Cl$	Isotopic shift	$\nu^{35}Cl$	$\nu^{37}Cl$	Isotopic shift
863.5475	863.5475	0.0	3176.6230	3176.6230	0.0
885.1836	885.1836	0.0	3177.3117	3177.3117	0.0
889.4835	889.4835	0.0	3177.5081	3177.5081	0.0
893.3492	893.3492	0.0	3177.8697	3177.8697	0.0
894.0575	894.0575	0.0	3178.4016	3178.4016	0.0
903.2597	903.2597	0.0	3178.4261	3178.4261	0.0
905.4736	905.4736	0.0	3197.0210	3197.0210	0.0
910.2435	910.2435	0.0	3197.2575	3197.2575	0.0
912.4084	912.4084	0.0	3197.5649	3197.5649	0.0
914.3097	914.3097	0.0	3197.7887	3197.7887	0.0
916.5828	916.5828	0.0	3198.1268	3198.1268	0.0
921.8187	921.8187	0.0	3198.4519	3198.4519	0.0
969.8975	969.8975	0.0			
